# Interactive network visualization of opioid crisis research: a tool for reinforcing data linkage skills for public health policy researchers

**DOI:** 10.3389/frai.2024.1208874

**Published:** 2024-04-05

**Authors:** Olga Scrivner, Thuy Nguyen, Michael Ginda, Kosali Simon, Katy Börner

**Affiliations:** ^1^Luddy School of Informatics, Computing, and Engineering, Indiana University, Bloomington, IN, United States; ^2^Rose-Hulman Institute of Technology, Terre Haute, IN, United States; ^3^School of Public Health, University of Michigan, Ann Arbor, MI, United States; ^4^O'Neill School of Public and Environmental Affairs, Indiana University, Bloomington, IN, United States; ^5^National Bureau of Economic Research, Cambridge, MA, United States

**Keywords:** public health policy, network visualization, data exploration, data linkage, data visualization, skills

## Abstract

**Background:**

Public health policy researchers face a persistent challenge in identifying and integrating relevant data, particularly in the context of the U.S. opioid crisis, where a comprehensive approach is crucial.

**Purpose:**

To meet this new workforce demand health policy and health economics programs are increasingly introducing data analysis and data visualization skills. Such skills facilitate data integration and discovery by linking multiple resources. Common linking strategies include individual or aggregate level linking (e.g., patient identifiers) in primary clinical data and conceptual linking (e.g., healthcare workforce, state funding, burnout rates) in secondary data. Often, the combination of primary and secondary datasets is sought, requiring additional skills, for example, understanding metadata and constructing interlinkages.

**Methods:**

To help improve those skills, we developed a 2-step process using a scoping method to discover data and network visualization to interlink metadata. Results: We show how these new skills enable the discovery of relationships among data sources pertinent to public policy research related to the opioid overdose crisis and facilitate inquiry across heterogeneous data resources. In addition, our interactive network visualization introduces (1) a conceptual approach, drawing from recent systematic review studies and linked by the publications, and (2) an aggregate approach, constructed using publicly available datasets and linked through crosswalks.

**Conclusions:**

These novel metadata visualization techniques can be used as a teaching tool or a discovery method and can also be extended to other public policy domains.

## 1 Introduction

The U.S. opioid epidemic is a major national concern. As of April 2023, the 12-month counts of reported deaths from drug overdose have increased by an estimated 6.5% compared with the year 2021—rising from 99,782 to 106,275 deaths (Ahmad et al., 2023). Particularly, the overdose involving synthetic opioids increased from 17 to 21.8% from 2020 through 2021, according to the National Center for Statistics (Spencer et al., 2022). Furthermore, among the 40.7 million people with a substance use disorder, only 1.1% (447,000) received treatment, 2.1% (837,000) felt the need but did not get treatment, and 96.8% felt no need for treatment (Substance Abuse and Mental Health Services Administration (SAMHSA), 2022). Some of the commonly reported reasons for not seeking treatment were (1) no health coverage, (2) not finding the program, (3) the perception of negative effects on their jobs, and (4) not knowing where to go.

To address the current opioid crisis, the Department of Health and Human Service (HHS)'s strategic priorities includes improvements in (1) pain management, (2) prevention, treatment, and recovery, (3) data and research related to the opioid crisis, and (4) overdose-reversing drugs (Price, 2017). It is crucial to integrate a holistic approach across multiple data resources, covering drug policy, pharmacy claims, treatment workforce, and opioid-related harms, among other research topics. Identifying trends and insights in this complex data presents a challenge and requires data analysis skills. Network visualization has been shown as a useful technique for analyzing complex relations in medical care services, clinical data, and physician network (Niyirora and Aragones, 2020). Many network models have included co-occurrences of records (e.g., patient diagnoses), chronological sequential occurrences (patient's admissions or discharge), and source and target occurrences (e.g., patient transfers). A recent study has extended network visualization to opioid prescription data, providing insights for healthcare professionals on the inappropriate use of drugs (Hu et al., 2020). While data analytical skills have been increasingly introduced to health policy programs, there is still a gap in learning network analysis skills (Payán, 2021).

Similarly, there is a growing body of systematic reviews and scientometric meta-analyses focusing on relationships between opioid use disorder and various factors, such as chronic pain, intervention, mitigation strategies, and policies (Chou et al., 2021; Gamage et al., 2023). Furthermore, a systematic meta-analysis of datasets unveils additional insights into available resources and their interlinkages. Stakeholders and practitioners are often challenged by the large number, complexity, and peculiarities of the existing data. Researchers may also not be aware of available resources as they are provided by many different organizations and have varying data quality and coverage. Some datasets are freely available, while others require the signing of legal documents or payment of fees for additional fields. Furthermore, some datasets are massive in size, requiring database expertise to run queries; others exist only as textual data in a PDF format and require pre-processing skills before usage. Providing data meta-analysis has become a new “informational asset” transforming how we observe and analyze data (Weber et al., 2014). In addition, linking resources together (or crosswalks) allows researchers and stakeholders to identify new areas for public or health interventions and provide evidence-based guidelines for practitioners and patients (Smart et al., 2018).

There is a growing need for metadata skills to help develop data strategies for identifying and linking resources. We propose a two-step framework to facilitate metadata discovery and relationships between datasets using metadata network visualization. In the first step, we show how to gather relevant metadata using the modified systematic review method. In the second step, we use a network design to represent linked datasets that communicate temporal, geospatial, and topical coverage via metadata nodes. This metadata visualization provides an alternative way to identify and integrate opioid-related datasets.

## 2 Background

The causes, consequences, and manifestations of the U.S. opioid crisis have been studied from many different angles, including prevention, treatment, drug prescription, law enforcement, criminal justice, and overdose reversal. Treatment expansions and prescription reductions are two essential steps in reducing mortality and improving safety for patients with chronic pain. Monitoring and regulatory policies play an equally important role in balancing between harms, cost, availability, and benefits of opioid use, as seen in policies such as prescription drug monitoring programs (PDMPs), health insurance expansions, and comprehensive federal legislation (e.g., the Comprehensive Addiction and Care Act) (Poitras, 2018; Scrivner et al., 2020). These efforts have led to a decrease in the overall U.S. drug prescription rate, from 81.3 per 100 people in 2012 to 46.7 in 2019 (CDC, 2019). But while the U.S. has had success in implementing these preventative measures, there has been an increase in harm from illicit drug sources, and there has been a challenge in improving treatment access for those suffering from addiction disorders. A major gap remains between service demand and supply: 94% of people aged 12 or older with a substance use disorder did not receive any treatment, according to the 2021 National Survey on Drug Use and Health data (NSDUH). The 2020 report on admissions to substance use treatment facilities (TEDs) has also reported a decrease in opioid-related admissions (381,040), as compared to 677,296 admissions in 2018. In terms of the number of facilities, only 1,754 out of 16,066 treatment facilities are Opioid Treatment Program (OTP) certified (Substance Abuse and Mental Health Services Administration, 2021). The 2021 County Business Patterns data (CBP) identifies 14,461 Substance Use Disorder Treatment (SUDT) outpatient centers, 44,731 Residential SUDT facilities, and 795 SUDT hospitals (API link—https://data.census.gov/table?q=CBP2021.CB2100CBP&n=62142:6222:6232&tid=CBP2021.CB2100CBP). Despite the high priority for training expressed by the U.S. Department for Health and Human Service and high job demand, the historical behavioral health (integrated mental and substance use disorder) workforce shortage has been a major roadblock (Skillman et al., 2016; McNeely et al., 2021).

The interdependence of these social, health, economic, and public policy factors calls for an interdisciplinary holistic and systematic approach where researchers and practitioners can zoom out and examine the problem as a whole and then zoom in to solve the most pressing issues that have the highest positive impact on improving health and services while decreasing crime and addictions-related disorders. One of the approaches is to provide a systematic review of opioid-related studies along with the secondary data relevant to the research (Leece et al., 2019; Maclean et al., 2020; Smart et al., 2020). This perspective enables researchers to discover data, identify new connections (linkages) between existing data, and learn about data accessibility and coverage. These systematic review studies also offer various perspectives on grouping datasets. From the economic perspective, data can be classified into several data categories relevant to understanding the opioid crisis: (1) pharmaceutical industries and medication prescriptions, (2) healthcare providers and labor market, (3) harms and crime, and (4) policies (Maclean et al., 2020). From the treatment perspective, data can be grouped by (1) intervention variables (e.g., prevention, treatment, and harm reductions) and (2) enabling variables (e.g., surveillance and stigma) (Leece et al., 2019). From the strategic perspective, data can be categorized according to the Health and Human Service strategic priorities: (1) better pain management, (2) addiction prevention, treatment, and recovery service, and (3) better targeting of overdose-reversing drugs (Smart et al., 2018, 2020). In addition, data can be classified based on type and format: national surveys, electronic health records (EHR), claims data, mortality records, prescription monitoring data, contextual and policy data, and others (national, state, local) (Smart et al., 2018, 2020).

In recent years, it also became common to share data with metadata, including data description, coverage, and attributes (Wu et al., 2023). As each dataset provides its unique identifier (e.g., geographical units, drug names, occupation, or billing codes), it is essential to (1) identify a crosswalk, an identifier that can link records to other datasets; (2) data coverage, such as number of records, frequency of updates, and data interval or data units (e.g., monthly and quarterly); and (3) data accessibility (e.g., open data or contract data). Understanding linkages and metadata becomes even more critical as many new datasets are released (Shlomo, 2018; Blanco et al., 2021). There are various ways to represent metadata, e.g., a tabular format or a dictionary schema. This representation, however, does not include the assessment of data coverage, its weaknesses, or its strengths. Novel solutions are offered by recent systematic dataset overviews: (1) each variable is provided with its relative frequency of occurrence in the reviewed literature (Leece et al., 2019); (2) a plus/minus sign is used to indicate strengths and weaknesses for each dataset (Smart et al., 2020); and (3) a “probabilistic linkage”, focusing on a visual representation of potential biomedical sources and the values of their linkages (Weber et al., 2014). The latter approach involves the use of a tabular form with sizes, shapes, colors, and positions to indicate data quality, data linkage, types of data (e.g., pharma, claims, EHR, and non-clinical data), data coverage, and even the probabilities for obtaining new data or linking existing data.

Furthermore, recent work on data integration and federation demonstrates advances in ontology and knowledge graph-based approaches allowing for integration, querying, analysis, and visualization across heterogeneous data sources (Sima et al., 2019; SN SciGraph, 2019; Cox et al., 2020; Amer-Yahia et al., 2021; Morris et al., 2023). For example, SPOKE (Morris et al., 2023) and Springer Nature SciGraph (SN SciGraph, 2019) use a knowledge graph (KG) to interlink and query different datasets. The SPOKE KG interlinks more than 30 publicly available biomedical databases, whereas SciGraph interlinks funders, projects, publications, citations, and scholarly metadata in support of data exploration. In addition, a natural language querying and visualization tool for biological knowledge is implemented for heterogeneous data sources (Sima et al., 2019). The INODE project (Amer-Yahia et al., 2021) incorporates machine learning techniques in support of guided, natural language querying and visualization of semantically integrated data sources in bio-medicine, astrophysics, and public policy.

Therefore, the deployment of visualization techniques emerges as a powerful data discovery tool and can be used to communicate metadata (data coverage, quality, and linkage). In addition, the graph representation not only facilitates a more intuitive understanding of complex datasets but also provides a unique resource to illustrate interlinkages between heterogeneous datasets, offering a more insightful perspective for data analysis than traditional data repositories.

## 3 Methods

To build metadata network visualization, we designed a two-step process enabling data discovery and synthesis in the first step and network graph implementation in the second step. This methodical process is not only reproducible but also adaptable, allowing for its application across various datasets and topics, thereby extending its utility and scope in diverse research contexts.

### 3.1 Data collection

Several recent systematic reviews on opioid-related studies include primary and secondary data. To collect metadata, we applied the modified scoping method by extracting and filtering studies from these systematic reviews. To represent a diverse collection of existing datasets, we included the following perspectives: (1) an economic systematic review with opioid-related datasets (Maclean et al., 2020), (2) pain management (Phillips et al., 2017), and (3) data sources for research and evaluation to address the Department of Health and Human Services (HHS) strategy combating opioid crisis (Smart et al., 2018, 2020). In addition, we parsed the scoping review references describing over 100 major economic studies on the U.S. opioid crisis (Maclean et al., 2020).

**Figure 1:**
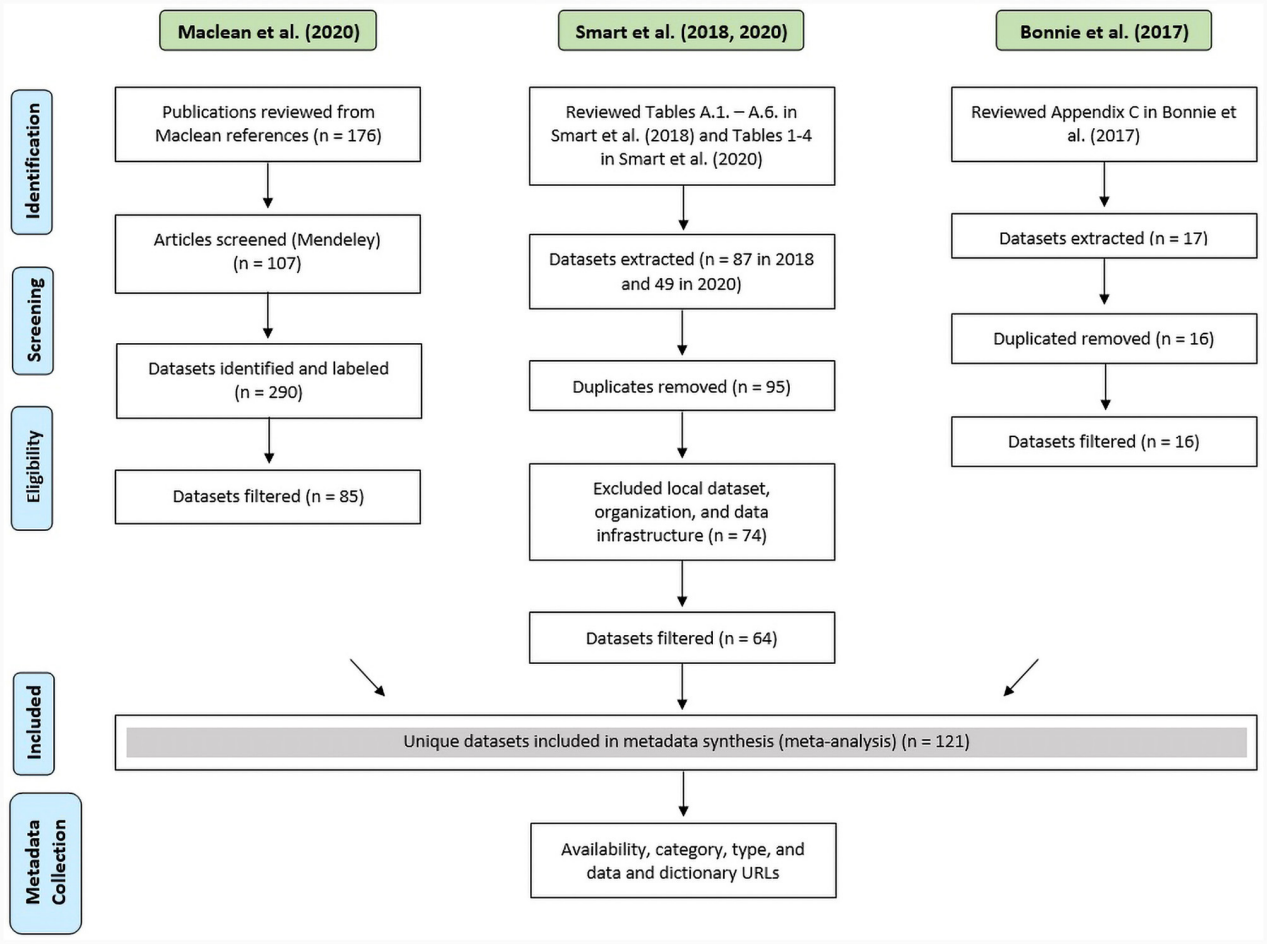
PRISMA flow diagram of the reference review process to identify datasets and data synthesis. Green labels are review studies, and blue labels are data collection steps.

Using the scoping review protocol (Arksey and O'Malley, 2005), we identified 176 cited papers (see Figure 1). Specifically, we established the following pipeline: (1) Importing—we imported the 176 cited articles ranging from 1986 to 2020 to the bibliographical software Mendeley. (2) Scanning—each article was scanned for datasets mentioned in the methodology section and articles without datasets were discarded. (3) Tagging—the remaining set (107 articles) was tagged with dataset names as they were used in the studies. As a result, we identified 283 unique name tags. Across the 107 studies, there were many inconsistencies in naming and spelling, for instance, “nvss,” “nvss multiple cause of death,” and “nvss multiple cause-of-death mortality” all referred to the U.S. mortality data from death certificates, produced by the National Center for Health Statistics. We normalized labels using OpenRefine and the Nearest Neighbor algorithm with Prediction by Partial Matching (PPM) distance (Stephens, 2018). The algorithm detected 61 clusters that were merged, resulting in 230 normalized labels. We manually inspected all labels and removed datasets that did not fit our eligibility criteria. The following filters were applied: (1) organization/agency sources without a reference to data (e.g., Bureau of Labor Statistics) [10 datasets removed], (2) only local data sources (e.g., Massachusetts All-Payer Claims Database) [21 datasets removed], (3) duplicate sources (e.g., Center for Disease Control and Prevention WONDER Multiple Cause of Death and National Vital Statistics System Multiple-Cause-of-Death files) [two datasets removed]. We classified the obtained datasets into the following categories as suggested by the policymakers experts we consulted: (1) pharmaceutical data–related to opioid prescription, (2) policy data–related to state drug laws, (3) opioid overdose data–related to treatment and treatment results, and (4) employment data–related to training and hiring in the substance use disorder treatment industry (SUDT). These datasets were then combined with the sources provided in Phillips et al. (2017) and Smart et al. (2020) summary tables. As a result, we identified 121 unique datasets extracted from prior scoping reviews for synthesis and data linkage exploration (see Table 1).

**Table 1:** Dataset format and category by availability.

	**Public**	**Non-public**	**Totals**
**Panel 1: dataset format**			
National surveys	17 (74%)	6 (26%)	23
Contextual and policy data	15 (94%)	1 (6%)	16
Claims and EHRs	8 (38%)	13 (62%)	21
Other	36 (59%)	25 (41%)	61
Totals	76 (63%)	45 (37%)	121
**Panel 2: dataset category**			
Harms	29 (62%)	18 (38%)	47
Jobs	22 (71%)	9 (29%)	31
Pharma	11 (41%)	16 (59%)	27
Policy	14 (88%)	2 (12%)	16
Totals	76 (63%)	45 (37%)	121

The non-public category includes private and data available by request.

### 3.2 Data synthesis

The data synthesis stage included gathering specific information from each identified dataset: (1) data description (dictionary, availability, category, and format) and (2) publication linkages. For each dataset, we assigned a format type, namely, national surveys, contextual data, and claims (Smart et al., 2018). National surveys are datasets that come from surveys conducted on a national sample. Contextual and policy data are datasets collected to analyze policy and policy changes. Claims and EHRs are datasets that include information on patient-level claims data for reimbursement and patients' health records. All remaining categories were grouped into *others*. The second taxonomy was the category, which is based on whether the dataset contains policy, pharmaceutical, opioid, or job-related data (see Tables 1, 2).

**Table 2:** Dataset category aggregated by three authors.

**Dataset category**	**Smart**	**Maclean**	**Bonnie**	**Totals**
Harms	28 (39%)	31 (44%)	12 (17%)	71
Jobs	5 (15%)	29 (85%)	0 (0%)	34
Pharma	18 (51%)	14 (40%)	3 (9%)	35
Policy	11 (48%)	11 (48%)	1 (4%)	23
Totals	62 (38%)	85 (52%)	16 (10%)	163

In addition, for each dataset, we searched for a data download link and a dictionary, which provides valuable information about data content and format. For some datasets, one or both of the URLs were not available. As a result, we provide at least one URL for 113 datasets and both URLs for only 33 of the 121 datasets. We were also unable to compile the crosswalks between all 121 datasets as some data are not available publicly. As a result, we manually assigned the following attributes to datasets: (1) data description (dictionary, size, category, and time coverage), (2) data linkages, and (3) scholarly metadata (relevant publications). Size was determined as the number of records based on the most recent year and split into three commonly used set sizes: (1) small (<10,000), (2) medium-sized (between 10,000 and 1,000,000), and (3) large (1,000,000 or greater). Note that the choice of the split threshold was arbitrary and based on the row number instead of the storage size as we did not have access to physical copies for each dataset. Time coverage provides information on the year when the dataset became available and the most recent data available for download. Several data attributes are used to identify data linkages: geographical units (e.g., state and county) and standard crosswalks [e.g., the North American Industry Classification System (NAICS), Drug Name]. Finally, for each dataset, we identified three recent publications using the Web of Science to illustrate research results derived from that data—this is not meant to be exhaustive but rather to show a starting point for a researcher looking into a new dataset. In total, 16 variables are provided for each dataset: common abbreviation, full name, data description, dataset category, source URL, dictionary URL, the number of records per year (most recent), size, time coverage (year-start and year-end), size, geo units, crosswalks, and three publications. Given the data accessibility restrictions, we were unable to assign these attributes to private or restricted datasets. As a result and after consulting with health policy experts, we created a subset providing all 16 variables for each dataset (see Table 3).

**Table 3:** Datasets to support research on the opioid crisis.

**Dataset**	**Description**	**Category**
CDC mortality	CDC Opioid Overdose Rate	Harms
TEDS-A	Treatment Episode Dataset: Admissions	Harms
NHIS	National Health Interview Survey	Harms
NSDUH	National Survey on Drug Use and Health	Harms
NAMCS	National Ambulatory Medical Care Survey	Harms
AHRF	Area Health Resources Files	Jobs
N-SSATs	National Survey of Substance Abuse Treatment Services	Jobs
QCEW	Quarterly Census of Employment and Wages	Jobs
CPS	Current Population Survey	Jobs
CBP	County Business Patterns	Jobs
ACS	American Community Survey	Jobs
MEPS	Medical Expenditure Panel Survey	Pharma
Sunshine Act	Open Payments	Pharma
SDUD (Medicaid)	State Drug Utilization Data	Pharma
Medicare*	Medicare Part D Prescription Drug Event	Pharma
ARCOS*	Automated Reports and Consolidated Ordering System	Pharma
CDC Prescription	CDC Drug Prescription	Pharma
PDMP	Prescription Drug Monitoring Program	Pharma
PDAPS	Prescription Drug Abuse Policy System	Policy
NAMSDL	National Alliance for Model State Drug Laws	Policy

Twenty datasets are extracted from the review study (Maclean et al., 2020) datasets marked with * require a request submission prior to download.

### 3.3 Network visualization

Network visualizations are widely used to capture the relationship between entities (e.g., co-authorship networks or gene-disease networks). These visualizations represent entities as nodes and their connections as edges, arranged in layouts that depict the overall connectivity structure and clusters while minimizing edge crossings. Networks can be derived from tabular data, such as the creation of a co-author network from a dataset containing the information on papers and the respective authors per paper. Co-author links connect all authors who appear together in a paper, creating an undirected weighted network (Emmert-Streib et al., 2018). Furthermore, nodes and edges within these networks can be enriched with additional visual cues, such as color or size coding, to highlight supplementary attributes. This can include characteristics such as the number of papers, the number of citations, the year of first publication, publication sources, and thematic categorization, enhancing the information conveyed by the visualization.

We developed two network visualization prototypes offering various metadata discovery perspectives: (1) citation network, offering potential insights into the frequency of data usage, and (2) conceptual network, providing insights into data relevancy. The design included the following pipeline: (1) data transformation, (2) network layout, and (3) interactive deployment. First, we transformed the CSV file with 121 datasets (rows) and 13 attributes (columns) into two distinct files: a “nodelist” and an “edgelist.” The nodelist included an additional identifier for each dataset, which was utilized in the edgelist to illustrate the connections between datasets. For instance, the ACS dataset was assigned the ID “ACS,” while the papers were labeled “Maclean2020” and “Smart2020” (refer to Table 4 for details). Since the ACS dataset was referenced in both papers, we established linkages from ACS (as the source) to Maclean2020 (as the target) and vice versa. Similarly, we established linkages from ACS (as the source) to Smart2020 (as the target) and vice versa, given that the network is an undirected graph. The resulting network consisted of 125 nodes categorized into five distinct groups and featured 203 edges (the example is outlined in Table 5).

**Table 4:** Nodelist used in the presented network visualization, partial—only four of 13 attributes are shown.

**ID**	**Format**	**Category**	**Availability**
ACS	National surveys	Jobs	Public
Maclean2020	Author	Author	(blank)
Smart2020	Author	Author	(blank)

**Table 5:** Edgelist used in the presented network visualization.

**Source**	**Target**	**Network type**	**ID**	**Weight**
Maclean2020	ACS	Undirected	17	1
Smart2020	ACS	Undirected	82	1

**Figure 2:**
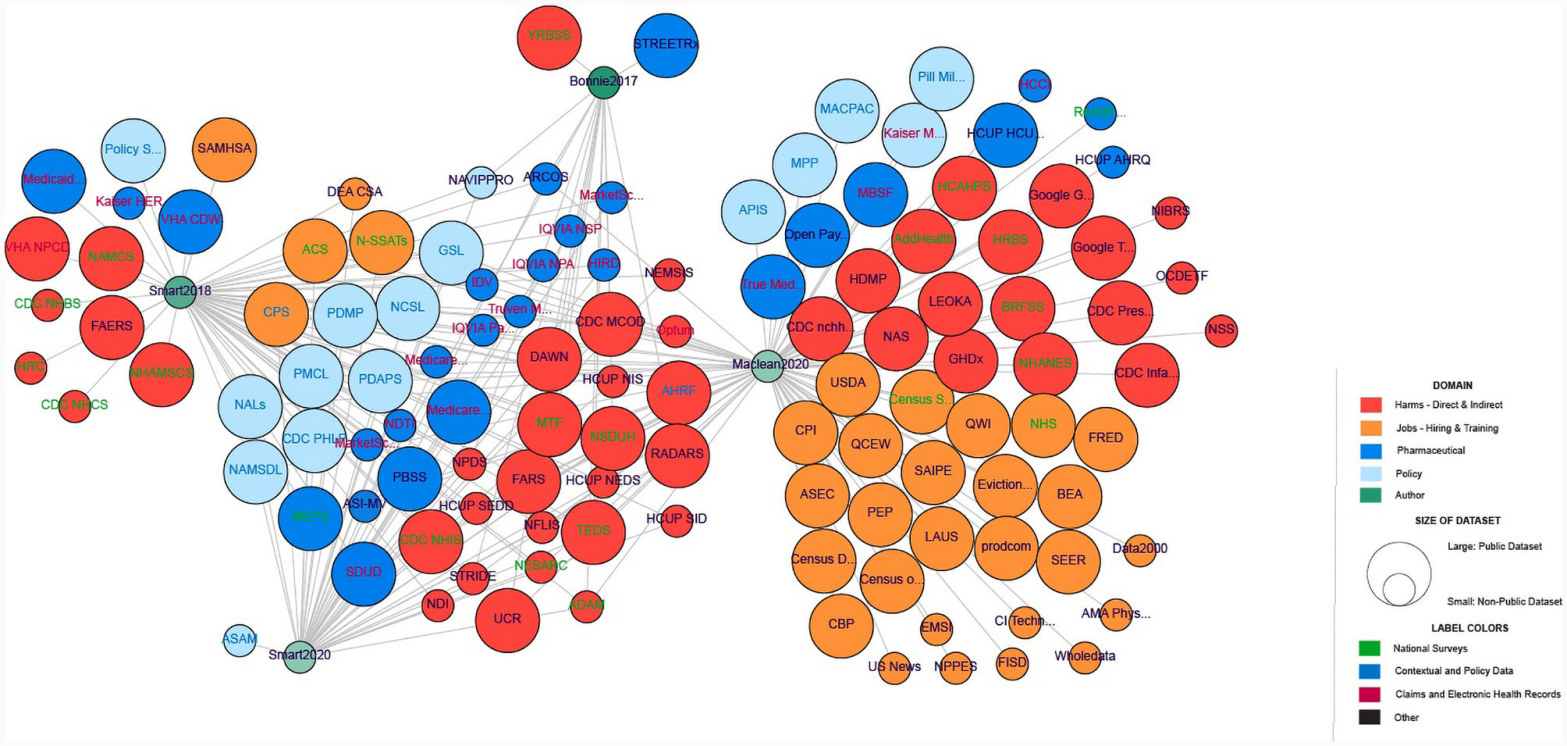
Network representation of the 121 datasets with policy data (in light blue), pharmaceutical data (dark blue), harms (red), jobs (hiring/training) data (orange), and authors (green). Transparency on the author nodes denotes the year of the publication (more transparency means a less recent year). Circle size corresponds to the availability (public or non-public) of the dataset. Label color denotes the type of dataset with national surveys (green), contextual and policy data (blue), claims and EHRs (maroon), and others (black).

**Figure 3:**
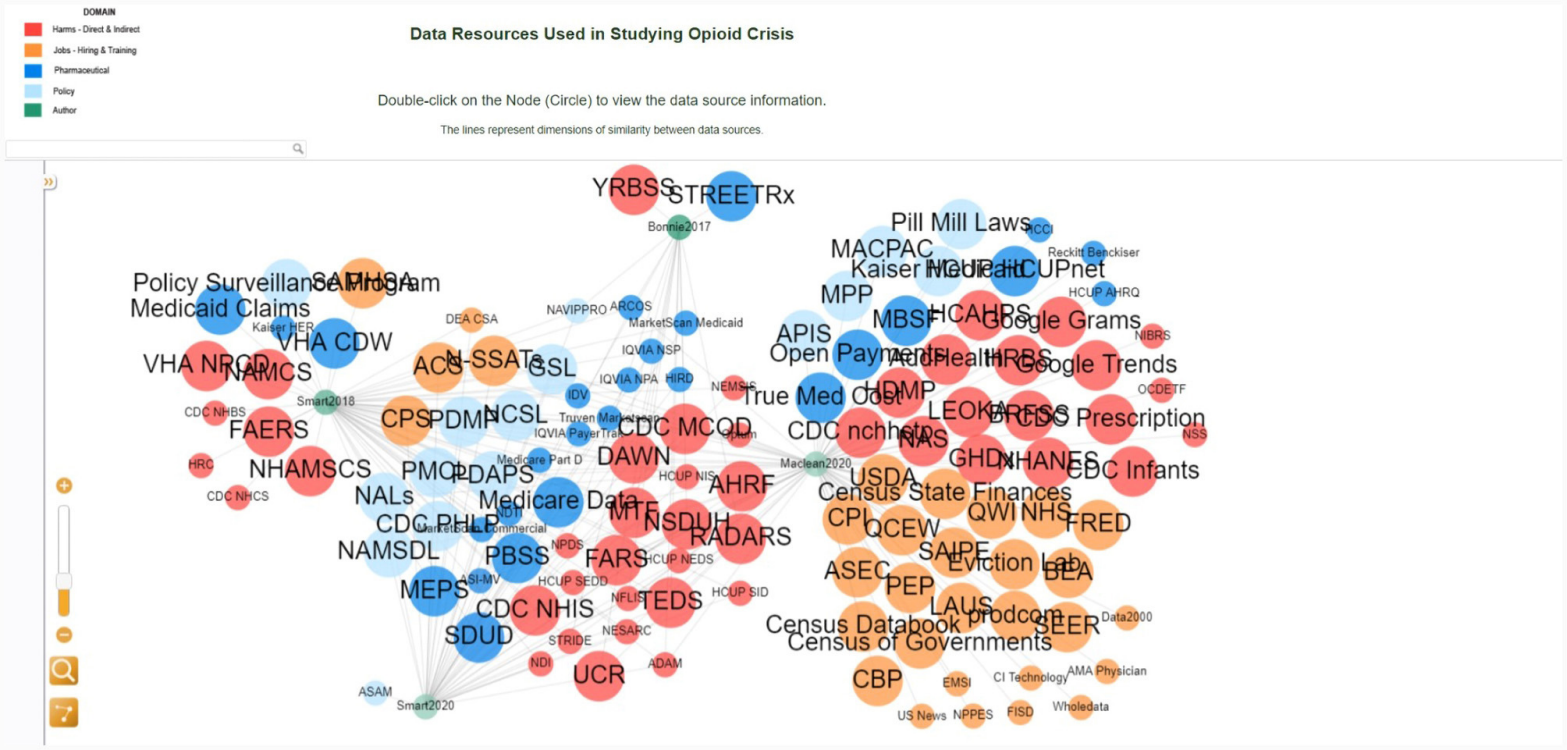
Interactive network visualization with the legend in the top left explaining color and size coding; details on demand in the lower left; interactive network layout on right.

Next, we used the Force Atlas 2 algorithm in Gephi (Bastian et al., 2009) (see Figure 2). Datasets were then grouped by paper and category with the 34 datasets mentioned in more than one paper being grouped in the middle. Datasets are also color-coded to visually render five categories: harms, jobs, prescription, policy, and author name. The workflow for creating this network in Gephi is available at GitHub (https://github.com/cns/iu/agc2/jobs). The interactive visualization was created using JavaScript GEXF viewer package (Velt, 2019). The Gephi network was exported from Gephi into a gexf format (.gexf), a native XML format suitable for JavaScript (js) interactive visualization frameworks. Then, the gefx.js code was updated and uploaded to GitHub. The interactive network is available at https://cns/iu.github.io/agc2/jobs/all_opioid_datasets/main/index.html and it supports search, filter, and details on demand (Shneiderman, 1996), as illustrated in Figure 3.

The second network prototype represents a conceptual linkage, following similar steps to create nodes and edges using geolocation and standard crosswalks as linkages. For instance, the CDC Mortality and TEDS share the same linkage attribute “State.” Thus, we can build their linkage from CDC Mortality with the ID “0” (source) to TEDs admission with the ID “1” (target) and vice versa since the network is undirected (see Tables 6, 7). The resulting network has 20 nodes of four categories and 146 edges of nine different types.

**Table 6:** Nodelist, partial—only six of 16 attributes are shown.

**ID**	**Label**	**Category**	**Size**	**Start**	**End**
0	CDC Opioid Mortality	Harms	S	1999	2018
1	Treatment Episode Dataset: Admissions	Harms	L	1992	2017
2	National Health Interview Survey	Harms	M	1963	2019

**Table 7:** Edgelist.

**Source**	**Target**	**Network type**	**ID**	**Label**	**Weight**	**Relation type**
0	1	Undirected	0	State	1	Geo unit
0	3	Undirected	1	County	1	Geo unit
0	5	Undirected	2	State	1	Geo unit

**Figure 4:**
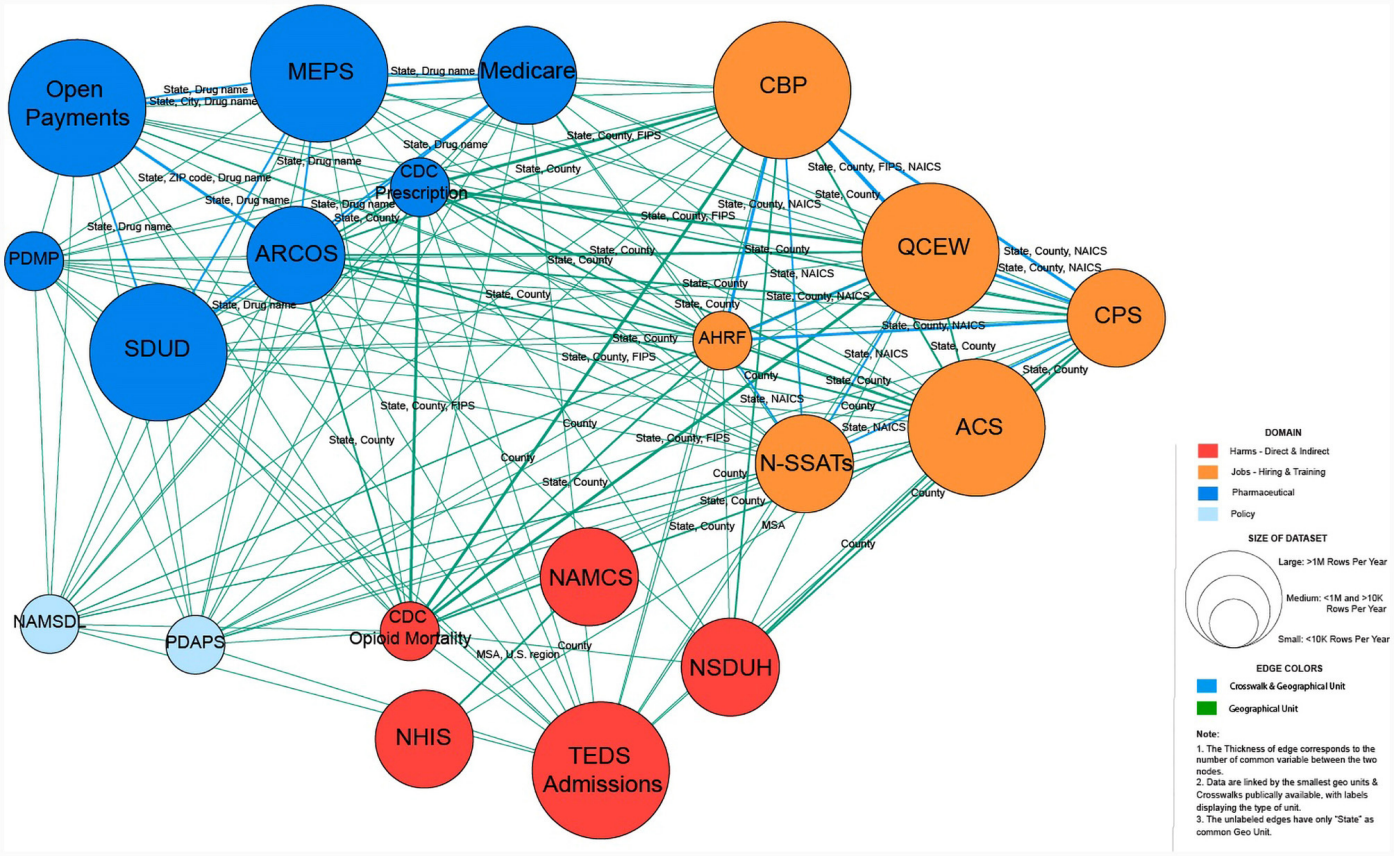
Network representation of the subset datasets with policy data (in light blue), pharmaceutical data (dark blue), opioid data (red), and jobs (hiring/training) data (orange). Circle size corresponds to the size of the dataset. The edge color denotes the type of linkage.

The second network is also color-coded to visually render four categories: harms, jobs, pharmaceuticals, and policy (see Figure 4). The workflow for creating this network in Gephi is available at GitHub (https://github.com/cns-iu/agc2-jobs). We followed the same steps as described earlier. The interactive network is available at https://cns-iu.github.io/agc2-jobs/20_datasets-main/index.html.

## 4 Discussion

**Figure 5:**
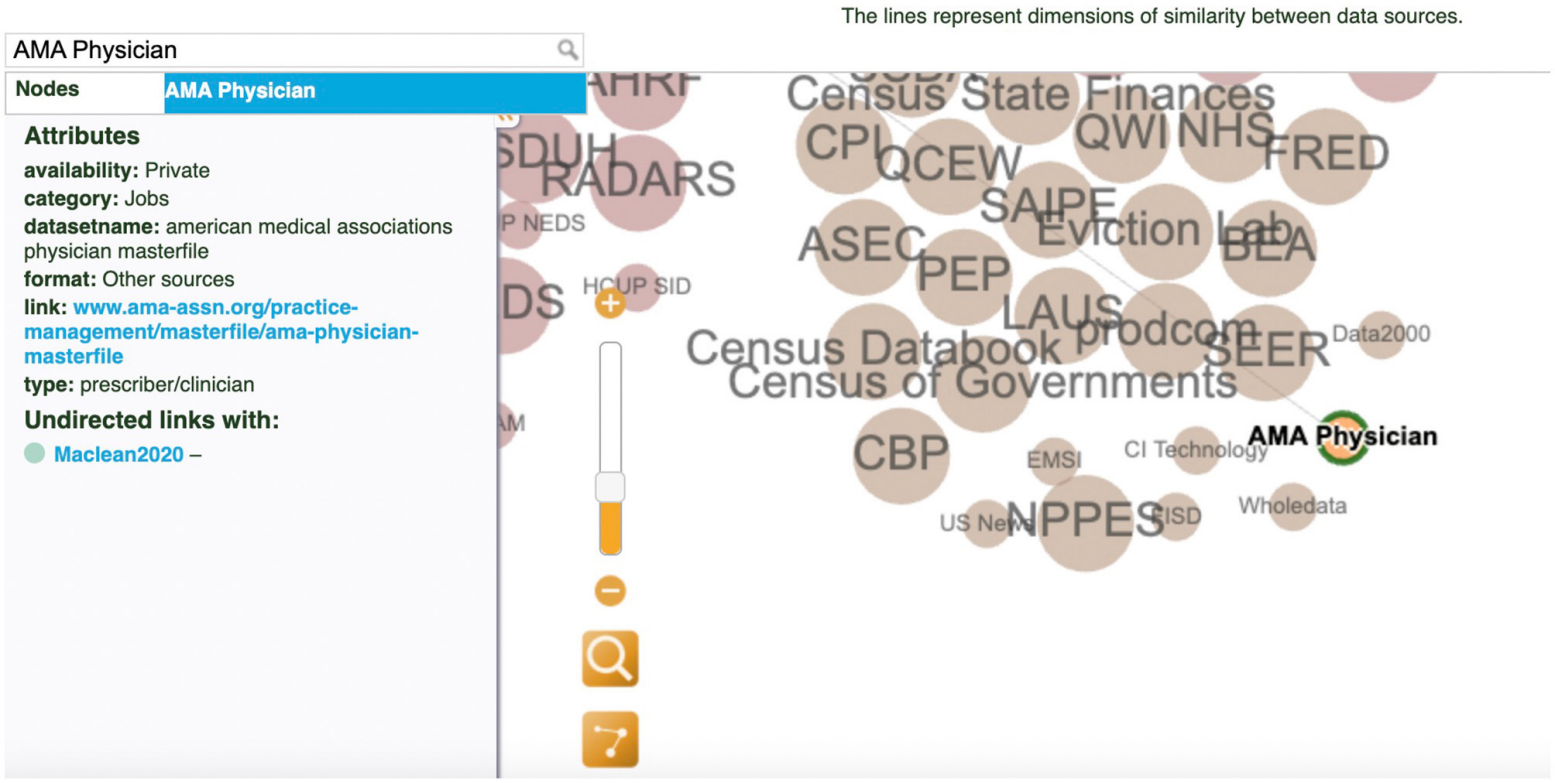
Search filter and information panel: The AMA Physician dataset.

The interactive visualization enables researchers to explore the relationships between data sources in the diverse context of public policy, economy, and treatment research related to the U.S. opioid crisis. In addition, it has the potential to help with data strategies and decision-making. For example, the user can search for a dataset AMA Physician by typing the name of the dataset in the search window or clicking directly on the node (see Figure 5). The information panel on the left displays the metadata information, showing that the dataset is private and related to the jobs category. It also provides the full dataset name, link, and the citation source. The researcher can also learn about other secondary datasets from the same job category color-coded in yellow, such as the NPPES dataset, which is publicly available. This information could help the researcher to make an informed decision on which dataset to use or to brainstorm new perspectives on opioid-related policy research.

**Figure 6:**
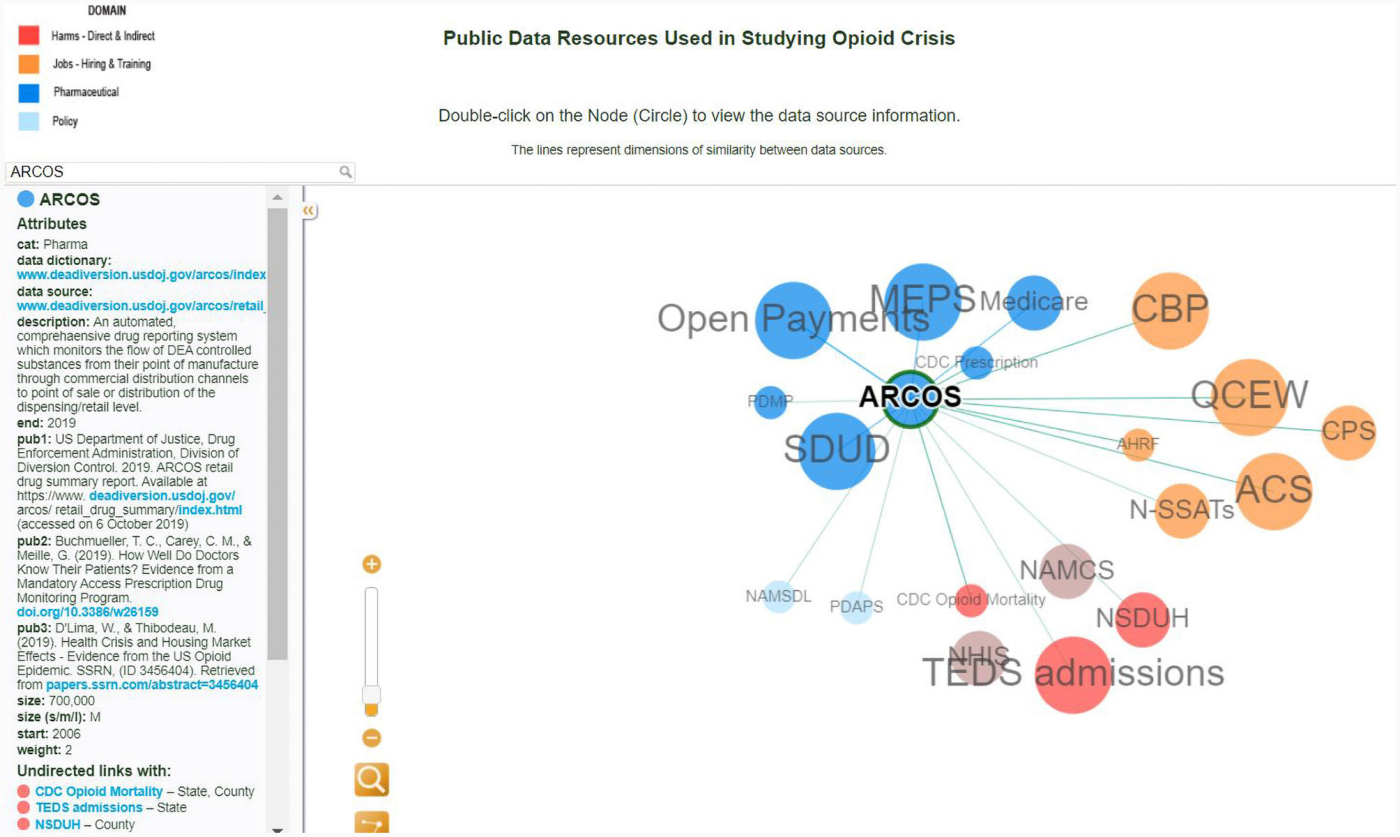
Interactive conceptual network visualization: the interlinkages for the ARCOS dataset.

Furthermore, the network representation demonstrates the potential for data discovery using metadata and data interlinkage skills as well as the discovery of the scholarly literature and case studies that use these datasets, which can assist researchers in identifying publicly available datasets, determining how these data can be combined in analysis, and surfacing relevant information about the provenance, availability, and definitions of data sources and variables. Figure 6 illustrates the discovery of the dataset ARCOS.

The node's dark blue color specifies that this dataset belongs to a pharmaceutical category. Clicking on the ARCOS node reveals links to the data dictionary and data source. In addition, the researcher is provided with a starter kit of the three most cited publications with this dataset. The size of the dataset is another useful attribute that allows the researcher to make decisions on storage space. Furthermore, this dataset could be linked at the state level with the CDC Mortality and TED Admissions data, using the drug name it can be connected with Open Payment and Medicare. This interactive network visualization of data relevant to public policy analysis may also be used as an instructional tool to help develop novel questions and assist in research. For example, by discovering the state linkage between ARCOS, CDC Mortality, and TED Admissions, the new question could be what states have the highest mortality rate as well as the highest pharmaceutical sales.

## 5 Conclusion

In alignment with the key priority set forth by the Department of Health and Human Services (HHS) to address the opioid crisis–enhancing information accessibility and promoting data-driven policy-making– our efforts have centered on metadata skills to discover and interconnect existing datasets. We developed a two-step process showing how to collect datasets using the scoping review method and transform data into network graphs. We curated 121 datasets, drawing from recent systematic reviews related to policy and opioid research. Furthermore, we have designed innovative visualization tool prototypes to assist researchers in data exploration. The interactive network visualization allows potential data users to navigate each dataset via data linkages, embedded data dictionaries, and recent publications using the selected dataset. A dataset can be identified as a complementary dataset to their current datasets to conduct relevant health services research studies or policy evaluations. Another data source can be used as an alternative dataset to validate their current data analysis. In addition, we provided the protocol for metadata collection and guidelines for network visualization and made it available for researchers to develop their dataset linkage networks.

This study has several limitations. First, we included datasets from four systematic review studies, potentially overlooking less common datasets. Second, we designed the linkage network between datasets only for 20 publicly available datasets, and we faced difficulties accessing metadata for non-publicly available datasets. This study also focuses on linkages at the concept level, rather than individual-level linking of data across datasets. This is an area of future development as it involves identifiable data and requires special data privacy considerations. Going forward, the same methodology can be applied to individual-level linked data and non-public resources. Another important area for future work is conducting user studies to identify how to best improve the visualization for different stakeholder groups and what additional datasets should be added. Finally, due to the limitation of dataset coverage, the current study assigned datasets only into four categories (policy, pharmaceutical, opioid, and jobs/training), excluding other important topics. Future research should use a more comprehensive view based on the opioid ecosystem approach (Stein et al., 2023).

## Data availability statement

The raw data supporting the conclusions of this article will be made available by the authors, without undue reservation.

## Author contributions

All authors listed have made a substantial, direct, and intellectual contribution to the work and approved it for publication.
